# Impact of non-contrast-enhanced imaging input sequences on the generation of virtual contrast-enhanced breast MRI scans using neural network

**DOI:** 10.1007/s00330-024-11142-3

**Published:** 2024-10-25

**Authors:** Andrzej Liebert, Hannes Schreiter, Lorenz A. Kapsner, Jessica Eberle, Chris M. Ehring, Dominique Hadler, Luise Brock, Ramona Erber, Julius Emons, Frederik B. Laun, Michael Uder, Evelyn Wenkel, Sabine Ohlmeyer, Sebastian Bickelhaupt

**Affiliations:** 1 Institute of Radiology, Universitätsklinikum Erlangen, Friedrich-Alexander-Universität Erlangen-Nürnberg (FAU), Erlangen, Germany; 2 Lehrstuhl für Medizinische Informatik, Friedrich-Alexander-Universität Erlangen-Nürnberg (FAU), Erlangen, Germany; 3 Institute of Pathology, Universitätsklinikum Erlangen, Erlangen, Comprehensive Cancer Center Erlangen-EMN, Friedrich-Alexander-Universität Erlangen-Nürnberg (FAU), Erlangen, Germany; 4 Department of Gynecology and Obstetrics, Erlangen University Hospital, Comprehensive Cancer Center Erlangen-EMN, Friedrich-Alexander-Universität Erlangen-Nürnberg (FAU), Erlangen, Germany; 5 Medizinische Fakultät, Friedrich-Alexander-Universität Erlangen-Nürnberg (FAU), Erlangen, Germany; 6 Radiologie München, München, Germany; 7 German Cancer Research Center (DKFZ), Heidelberg, Germany

**Keywords:** Magnetic resonance imaging, Breast imaging, Neural network, Artificial intelligence

## Abstract

**Objective:**

To investigate how different combinations of T1-weighted (T1w), T2-weighted (T2w), and diffusion-weighted imaging (DWI) impact the performance of virtual contrast-enhanced (vCE) breast MRI.

**Materials and methods:**

The IRB-approved, retrospective study included 1064 multiparametric breast MRI scans (age: 52 ± 12 years) obtained from 2017 to 2020 (single site, two 3-T MRI). Eleven independent neural networks were trained to derive vCE images from varying input combinations of T1w, T2w, and multi-b-value DWI sequences (b-value = 50–1500 s/mm^2^). Three readers evaluated the vCE images with regard to qualitative scores of diagnostic image quality, image sharpness, satisfaction with contrast/signal-to-noise ratio, and lesion/non-mass enhancement conspicuity. Quantitative metrics (SSIM, PSNR, NRMSE, and median symmetrical accuracy) were analyzed and statistically compared between the input combinations for the full breast volume and both enhancing and non-enhancing target findings.

**Results:**

The independent test set consisted of 187 cases. The quantitative metrics significantly improved in target findings when multi-b-value DWI sequences were included during vCE training (*p* < 0.05). Non-significant effects (*p* > 0.05) were observed for the quantitative metrics on the full breast volume when comparing input combinations including T1w. Using T1w and DWI acquisitions during vCE training is necessary to achieve high satisfaction with contrast/SNR and good conspicuity of the enhancing findings. The input combination of T1w, T2w, and DWI sequences with three b-values showed the best qualitative performance.

**Conclusion:**

vCE breast MRI performance is significantly influenced by input sequences. Quantitative metrics and visual quality of vCE images significantly benefit when multi b-value DWI is added to morphologic T1w-/T2w sequences as input for model training.

**Key Points:**

***Question*** *How do different MRI sequences impact the performance of virtual contrast-enhanced (vCE) breast MRI?*

***Findings*** *The input combination of T1-weighted, T2-weighted, and diffusion-weighted imaging sequences with three b-values showed the best qualitative performance.*

***Clinical relevance*** *While in the future neural networks providing virtual contrast-enhanced images might further improve accessibility to breast MRI, the significant influence of input data needs to be considered during translational research.*

**Graphical Abstract:**

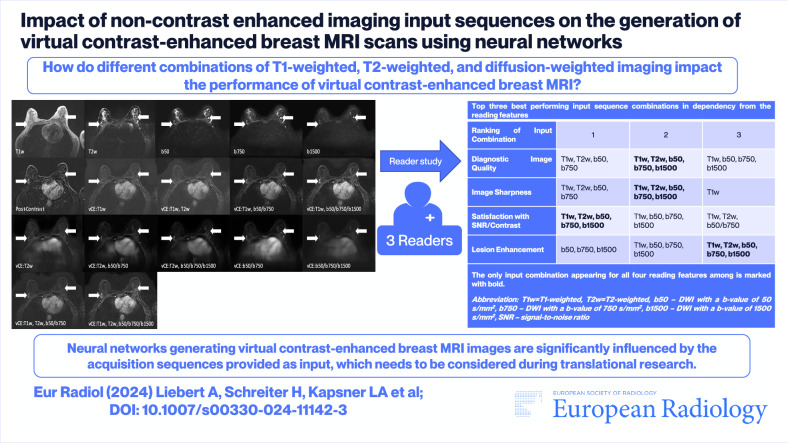

## Introduction

Breast MRI routinely includes the acquisition of contrast-enhanced (CE) image series [1] after intravenous injection of gadolinium-based contrast agents (GBCAs). GBCAs help contrast tissue changes associated with altered neoangiogenesis and/or extracellular GBCA distribution patterns frequently found in significant findings. However, the necessity of GBCA administration might limit accessibility, especially in screening settings [2–5]. GBCA administration increases both direct and indirect costs of breast MRI, challenging the cost-effectiveness during screening in low- and moderate-risk populations [2, 6, 7]. This is due to additive patient preparation time even with shortened MRI scanning time (e.g., in abbreviated approaches) [8, 9] and the costs of the GBCA itself [10–12]. Furthermore, gadolinium depositions in the human body have been described in the literature [13], potentially constituting a barrier to annual screenings.

The generation of virtual contrast-enhanced (vCE) breast MRI scans from unenhanced acquisitions using deep learning was recently shown to be technically feasible throughout several publications [14–18]. Given the novelty of this research field, a high variability is yet observed in the literature with regard to the choice of input data used to train vCE imaging neural networks. Various protocols have been employed, including T1-weighted (T1w) sequences alone [14, 15], combinations of T1w and T2-weighted (T2w) image acquisitions [16], and more complex protocols incorporating diffusion-weighted imaging (DWI) [17].

Our study aims to systematically investigate the impact of different MRI input sequences on the ability of neural networks to generate vCE breast MRI scans using quantitative global and regional metrics in both enhancing and non-enhancing findings. Additionally, a multi-reader study was conducted to investigate the image quality and lesion/non-mass enhancement (NME) conspicuity.

## Materials and methods

### Summary of patient cohort and MRI protocol

The ethics committee of Friedrich-Alexander University Erlangen-Nürnberg approved this retrospective study and waived the need for informed consent. The study included 1064 clinically indicated routine breast MRI scans (age 52 ± 12 years) acquired between 01.2017 and 06.2020 at University Hospital Erlangen. Examinations were performed in the prone position, using two 3-T MRI scanners (MAGNETOM Skyra-Fit. MAGNETOM Vida, Siemens Healthineers) and a separate 18-channel breast coil (both Siemens Healthineers) dedicated for each of the scanners. The examination protocol included T1w, T2w fat-saturated (further referred to as T2w), multi-b-value DWI (b-values: 50, 750, 1500 s/mm^2^), and five-timepoint dynamic contrast-enhanced (DCE) image acquisitions with subtraction series automatically derived on the MRI scanner. Further sequence details are given in Table 1.

**Table 1:** MRI protocol

Sequence	Sequence type	Matrix size	FoV (mm × mm)	Slice thickness (mm)	TR (ms)	TE (ms)	IR (ms)	FA (°)	No. of averages	Scan duration	Additional parameters
T1w^a^	3D-GRE	448 × 448 × 112–128	360 × 360–430 × 430	1.5–1.8	5.97	2.46	–	10	1	Approx. 1 min/1 min 10 s^d^	Grappa 3/2^d^
T2w	2D-SE	448 × 448 × 34–49	340 × 340–430 × 430	4	3570–5020	60, 70	230	108	2	Approx. 3 min 45 s/1 min 54 s^d^	STIR Fat-saturation, Turbo factor 16/15^d^ Grappa 2/3^d^
DWI^b^	2D-IR-DWI-EPI	256 × 160–200 × 34–49	350 × 219–430 × 269	4	6290–9660	66, 70	220/250^d^	90	3/8/20 or 3/8/15^c^	Approx. 3 min 24 s/3 min 15 s^d^	STIR Fat-saturation Grappa 2/2

*FoV* field of view, *TR* repetition time, *TE* echo time, *IR* inversion recovery time, *DWI* diffusion-weighted imaging, *GRE* gradient echo, *SE* spin echo, *EPI* echo-planar imaging, *GBCA* gadolinium-based contrast agent, *STIR* short TI inversion recovery

^a^ Acquired before and during the five time points after intravenous GBCA injection (gadobutrol; Bayer, Leverkusen, Germany; 0.1 mmol/kg/body weight, injection speed = 2 mL/s). Acquisitions were performed in 60/70^d^ second intervals, with the acquisition of the DCE images starting 20/25^d^ seconds after the administration of the GBCA

^b^ DWI scan was acquired using three b-values: 50, 750, and 1500 s/mm^2^

^c^ No. of averages for DWI indicates the number of averages for each b-value. The first set of values was used in acquisitions performed on both Skyra-Fit and MAGNETOM VIDA and the second set was used only for acquisitions performed on MAGNETOM VIDA

^d^ Values presented refer to values for the Skyra Fit/ Vida scanner models

**Fig. 1:**
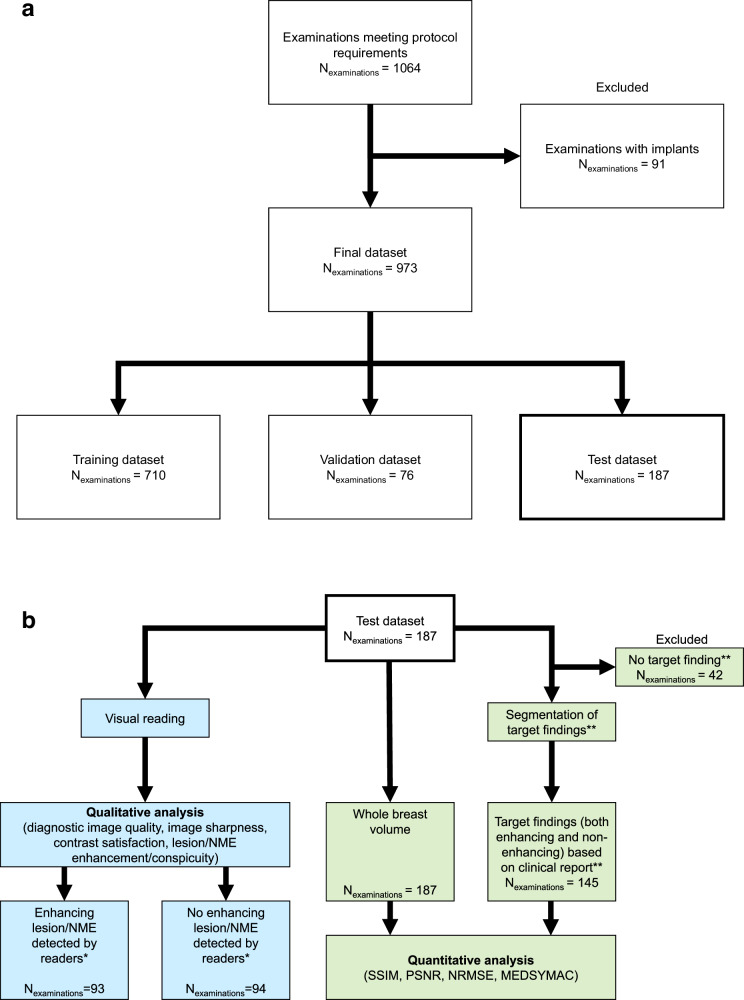
**a** The full cohort consisted of all patients who underwent diagnostic breast MRI on one of two clinical 3-T scanners (MAGNETOM Skyra-Fit or MAGNETOM Vida, Siemens Healthineers) from January 2017 to June 2022. The protocol included a five-point dynamic contrast-enhanced acquisition, a T1-weighted image acquisition, a T2-weighted fat-saturated image acquisition, and a multi-b-value diffusion-weighted image acquisition containing b-values of 50, 750, and 1500 s/mm^2^. **b** Flow chart of the two experiments conducted. Visual reading was performed by three independent readers. *Enhancing lesions/NMEs were stratified according to the majority vote of the three readers. **Target findings refer to the findings within the examination that could be both benign or malignant and non-mass or mass enhancement, as well as findings not enhancing (e.g., cysts) but morphologically delineated from healthy fibro-glandular tissue

Ninety-one MRI scans were excluded owing to the presence of breast implants (Fig. 1a). The final cohort (*n* = 973) was randomly divided at the patient level into training (*n* = 710), validation (*n* = 76), and separate independent test sets (*n* = 187).

The independent test set was evaluated in two experiments (Fig. 1b).

- Experiment 1: Quantitative analyses were performed for the full test dataset for (1) the entire breast volume (*n* = 187) and (2) using a subset of the examinations in which target findings, either enhancing or non-enhancing, were identified and subsequently manually segmented (145/187 cases), as described in the “Quantitative analysis” subsection.
- Experiment 2: Qualitative analyses were performed via visual readings of three independent readers using the full independent test set (*n* = 187) and corresponding scores described in the “Qualitative analysis” subsection.

All cohort examinations (*n* = 973) were previously included in studies focused on detecting or forecasting artifacts occurrence in DCE and DWI-derived maximum-intensity projections [19, 20].

### Input sequences used for vCE breast MRI

As our breast MRI dataset contained all three types of native input acquisitions (T1w, T2w, and advanced DWI) described in the literature, we investigated the use of all possible combinations of these inputs. However, for reasons stated in the Supplementary Material section “Rationale for the exclusion of data types,” we decided not to use scanner-derived ADC maps used by Chung et al [17] and simulated low-dose images used by Muller-Franzes et al [16]. The b-values of 50, 750, and 1500 s/mm^2^ from the advanced DWI (further called b50, b750, and b1500, respectively) were grouped as input channels of b50/b750 and b50/b750/b1500, thus reflecting clinically used combinations [21, 22]. To investigate the impact of the individual input combinations, we decided to investigate all possible combinations of the T1w, T2w, and the two DWI input sets. This resulted in the following combinations of non-contrast-enhanced input sequences:

1. T1w
2. T1w, T2w
3. T1w, b50/b750
4. T1w, b50/b750/b1500
5. T2w
6. T2w, b50/b750
7. T2w, b50/b750/b1500
8. b50/b750
9. b50/b750/b1500
10. T1w, T2w, b50/b750
11. T1w, T2w, b50/b750/b1500

The combinations including just T2w, just DWI sequences or the combinations of both were not previously proposed in other works. We included them into the evaluation for potential use in non-contrast-enhanced abbreviated protocols [23, 24].

### Neural network architecture and training

The two predominant architectures used in generating vCE breast MRI scans were generative adversarial networks (GANs) [14–16] and encoder–decoder convolutional neural networks [17, 18]. In this work, we employed a 2D-U-net architecture with three encoder and decoder stages, an established encoder–decoder network, for our analysis. The rationale for selecting the U-net architecture was based on its robustness compared with GAN-based solutions. By employing a simpler and more robust architecture, we aimed to minimize potential confounding factors introduced by more complex models, such as the necessity to optimize discriminator networks in GANs.

The 2D-U-Net architecture implementation, data preprocessing, and network training are detailed in the Supplementary Fig. 1 and Supplementary Material sections “Image preprocessing” “Neural network architecture and training.” During training, the respective combinations of the pre-contrast MRI acquisitions were fed slice-wise to the neural network. The second post-contrast subtraction of the dynamic contrast-enhanced series was selected as the ground truth.

### Quantitative analysis

To perform quantitative evaluation, manual segmentations of target findings in the independent test set were performed as laid out in detail in the Supplementary Material section “Segmentation of target findings.”

For quantitative evaluation of the image series, two similarity metrics: structural similarity index (SSIM) [25], peak signal-to-noise ratio (PSNR) and two error metrics: normalized root mean square error (NRMSE) and median symmetrical accuracy (MEDSYMAC) [26] were calculated. The evaluation was performed within the full breast volume and inside of the target findings.

Details on the statistical analysis of differences in the scores are provided in the Supplementary Material Section “Statistical analysis.” The rationale for the choice of evaluated metrics is detailed in the Supplementary Material section “Quantitative metrics choice.”

### Qualitative analysis

Similar to the work of Muller-Franzes et al [16], the reading task was performed as a multi-reader side-by-side comparison of vCE and CE images. In this reading, slices from the original CE image acquisition and the corresponding slice from all eleven vCE input combination variations were presented to the readers. All evaluations were performed by three independent readers (board-certified radiologists: S.B. and D.H., both with > 10 years of experience; medical research assistant: J.E., with > 2 years of experience in breast MRI) using multi-point Likert-like scales. The readers were blinded to the input sequences combination used to generate each virtual contrast. However, due to their expertise in breast MRI, the readers might have inferred some of the likely input combinations, e.g., vCE images created solely from DWI inputs retained a noticeable “DWI-like” texture. Furthermore, certain subtle yet discernible textural features in the CE images made them distinguishable from the vCE images, e.g., minor non-diagnostically significant but visible artifacts that are typically present in CE subtractions and a more blurred appearance of background parenchymal enhancement in the vCE images.

In order to allow a more accurately reflection of the variations in the different vCE input combinations a 11-point Likert-like scale was chosen as previously suggested [27]. During reading, the following features were evaluated for both CE and vCE images using an 11-point Likert-like scale (0-non-acceptable/insufficient, 10-excellent): diagnostic image quality, image sharpness, satisfaction with image contrast and visual signal-to-noise ratio. Further, potentially significant enhancing lesions or NMEs were compared with the surrounding tissue and evaluated using a 9-point Likert-like scale for lesion/NME conspicuity (0-lesion/NME not visible, 8-perfect lesion/NME conspicuity) on the CE subtraction images. This was done to allow comparing the Likert-scores to the vCE images, in which the readers were asked whether the enhancing lesion/NME was correspondingly reflected regarding its conspicuity using an 11-point Likert-like scale (0-lesion/NME not visible, 8-fully identical lesion/NME conspicuity compared with GBCA subtraction) with the two “additional scores” of 9-lesion/NME enhancing stronger than on CE subtraction images and 10-lesion/NME enhancing much stronger than on CE images being available during reading. Details on the statistical evaluation of the qualitative scores are provided in the Supplementary Material section “Statistical analysis.”

## Results

### Demographics and clinical findings

The demographics of the full data set and respective subsets are presented in Table 2. In the test cohort (*n* = 187), *n* = 67 (35.8%) cases had a malignant lesion (median size = 20.4 mm, 25th–75th percentile = 13.0–37.7 mm). Among the *n* = 67 malignant lesions, the following histopathology was described: *n* = 11 (16%) ductal carcinoma in situ (DCIS), *n* = 7 (10%) invasive lobular carcinoma (ILC), *n* = 1 mucinous carcinoma, *n* = 48 (72%) invasive breast cancer non-special type among *n* = 10/48 non-special type with associated DCIS.

**Table 2:** Demographics of the training/validation and independent test cohorts

	Entire dataset	Training	Validation	Independent test set
No. of patients	869 (100%)	606 (69.7%)	76 (8.8%)	187 (21.5%)
No. of examinations	973	710	76	187
Split ratio of examinations (%)	100	73	7.8	19.2
Age (years)	52 ± 12	50 ± 12	52 ± 12	51 ± 14
Routine BI-RADS score^a^				
0	23 (2.4%)	20 (2.8%)	0 (0%)	3 (1.6%)
1	3 (0.3%)	2 (0.3%)	0 (0%)	1 (0.5%)
2	461 (47.4%)	330 (46.5%)	42 (55.3%)	89 (47.6%)
3	73 (7.5%)	49 (6.9%)	7 (9.2%)	17 (9.1%)
4	114 (11.7%)	96 (13.5%)	4 (5.3%)	14 (7.5%)
5	70 (7.2%)	48 (6.8%)	3 (4.0%)	19 (10.2%)
6	229 (23.5%)	165 (23.2%)	20 (26.3%)	44 (23.5%)

^a^ The BI-RADS score refers to the highest BI-RADS score given for an examination during routine clinical reading; age is presented as means and standard deviations. Percentages of BI-RADS scores are given as the ratio of the respective dataset

Based on the clinical report, *n* = 145 (77.5%) of the examinations had a target finding as defined in the methods (median size = 14.8 mm, 25th–75th percentile = 7.1–26.4 mm).

### Experiment 1: Quantitative analysis

**Fig. 2:**
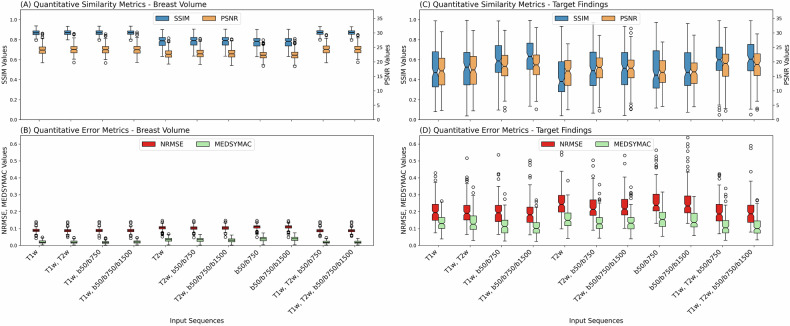
Box plots with notches for the quantitative similarity (SSIM and PSNR) and error (NRMSE and MEDSYMAC) metrics for the full cohort (**A** and **B**, respectively) and only the enhancing lesions (**C** and **D**, respectively). Notches indicate the 95% confidence intervals, boxes indicate the interquartile range, and the circles indicate the outlier values. A significant difference in the SSIM and PSNR could be observed in the breast volume between the input combinations with and without a T1w image acquisition. The similarity and error metrics were significantly higher and significantly lower in the target findings, respectively, when compared with the input sequence combinations, including both T1w image and DWI acquisitions and combinations that missed either of those acquisitions. T1w, T1-weighted; T2w, T2-weighted; b50, DWI acquisition with a b-value of 50 s/mm^2^; b750, DWI acquisition with a b-value of 750 s/mm^2^; b1500, DWI acquisition with a b-value of 1500 s/mm^2^; SSIM, structural similarity index; PSNR, peak signal-to-noise ratio; NRMSE, normalized root mean square error; MEDSYMAC, median symmetrical accuracy

Figure 2 shows the technical performance metrics of all eleven input combinations in the entire breast volume and segmented findings. Significant differences were found in all quantitative scores using the Friedmann test for both breast volume evaluation and target finding evaluations (all *p* < 0.001). The means and standard deviations of the quantitative metrics and detailed post-hoc Nemenyi test results of multiple comparisons of the input sequence performances are presented in Supplementary Fig. 2.

In the breast volume similarity metrics increased significantly when T2w sequences were added to the T1w sequences alone (SSIM = 87.06 ± 2.57 vs 86.91 ± 2.58, *p* = 0.011; PSNR = 24.33 ± 1.79 vs 24.18 ± 1.82 dB, *p* = 0.011). However, while both error metrics decreased (NRMSE = 8.77 ± 1.17 vs 8.91 ± 1.20, *p* = 0.011; MEDSYMAC = 2.02 ± 0.92 vs 2.08 ± 0.95 dB, *p* = 1.0), only the NRMSE metric changed significantly. The T1w sequences alone yielded significantly higher similarity metrics than the T2w (*p* = 0.011) or both combination of the DWI (*p* = 0.011) alone as well as both combination of just these two sequences (*p* = 0.011). The full-breast volume similarity metrics did not significantly differ neither when the b50/b750 combination nor when the b50/b750/b1500 combination (SSIM = 87.00 ± 2.55, *p* = 1.0, PSNR = 22.82 ± 1.70 dB, *p* = 1.0) was added to the T1w sequence (SSIM = 87.00 ± 2.55 for the combination of T1w, b50/b750/b1500, *p* = 1.0) when compared to the input of just T1w sequence. Significant improvement in regards to the similarity metrics could be observed when both T2w and different combinations of DWI sequences were added to the T1w when compared to only using the T1w input sequence (*p* = 0.11 for both metrics and both comparisons).

When the metrics of the segmented target findings within the breast were considered, the SSIM significantly increased when multi-b-value DWI with ultra-high b-values of b1500 was added, from 50.25 ± 22.84 (T1w) and 51.94 ± 22.80 (T1w, T2w) to 63.08 ± 20.16 (*p* = 0.011) and the MEDSYMAC decreasing from 13.92 ± 5.19 (T1w) and 14.12 ± 6.28 (T1w, T2w) to 11.01 ± 5.07 (*p* = 0.011 for both). Adding T2w sequences to the combination of T1w, b50/b750/b1500 did not significantly influence the quantitative metrics (*p* = 1.0) in any evaluation. Further numerical results of all sequence combinations are shown in Supplementary Table 2.

### Experiment 2: Qualitative analysis

**Fig. 3:**
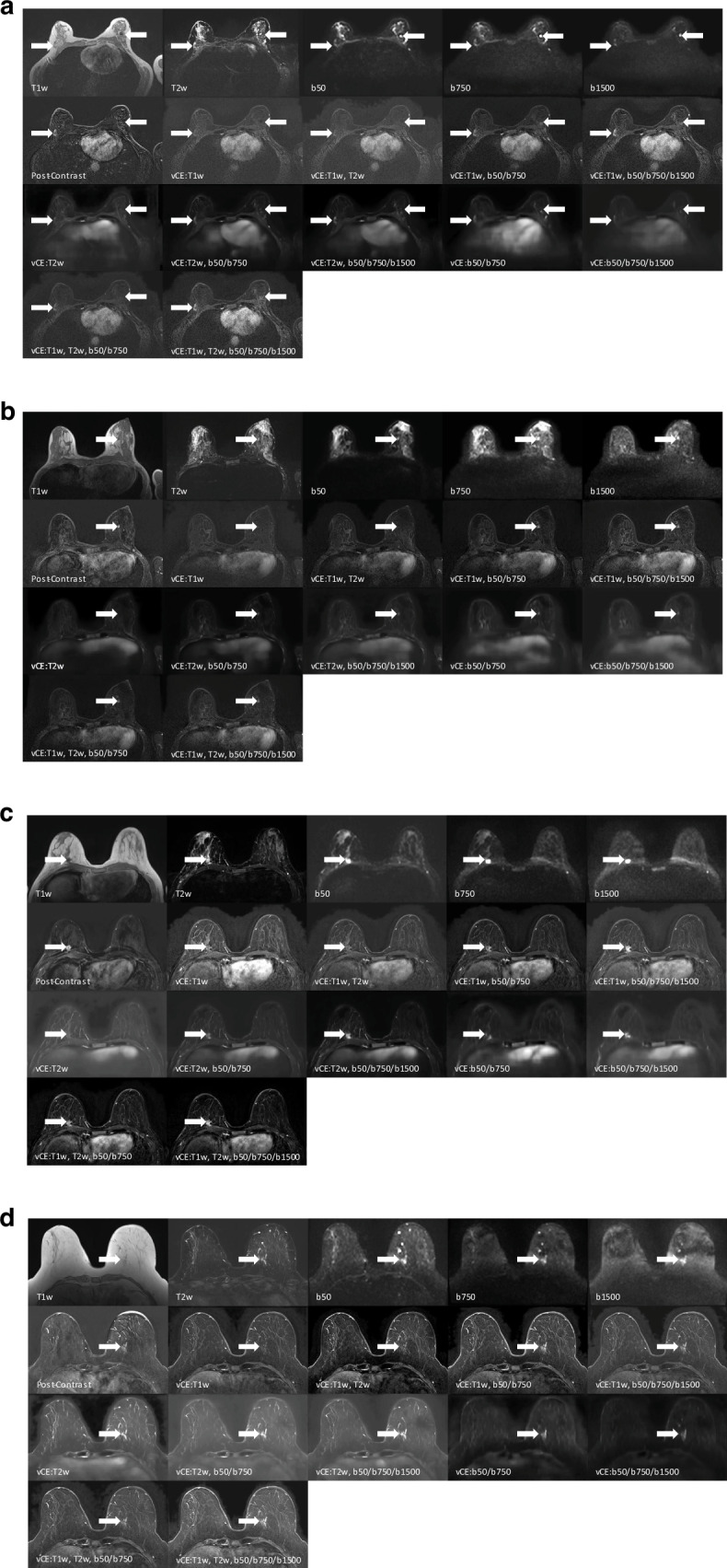
Diagnostic breast MRI slices that were read by the three readers showing the input sequences, post-contrast-enhanced image acquisition, and eleven virtual contrast-enhanced versions. **a** Scans of a 63-year-old patient show a lesion in the right breast (16.2 mm, white arrow) enhancing in the GBCA-enhanced post-contrast subtraction image (“Post-Contrast”). The lesion does not enhance in the vCE images that did not include any DWI sequences as input. Histopathology confirmed a breast carcinoma (NST) with DCIS (ductal carcinoma in situ). An additive finding of a complex cyst is demonstrated in this case in the left breast with a high signal in the b = 1500 s/mm^2^ b-value DWI and a heterogeneous signal in the T1w sequence. The virtual contrast-enhanced data does highlight the contrast-simulation in the carcinoma while the complex cyst does not simulate a contrast-agent uptake. **b** Scans of a 47-year-old patient show a lesion (5.7-mm, white arrow) enhancing in the GBCA-enhanced post-contrast subtraction image (“Post-Contrast”). The lesion does not enhance in the vCE images that did not include any DWI sequences as input. Histopathology confirmed DCIS (ductal carcinoma in situ). **c** Scans show a 40-year-old patient (13 mm, white arrow) with a lesion in the right breast enhancing in the GBCA-enhanced post-contrast subtraction image (“Post-Contrast”). The lesion barely enhances in the vCE images that did not include any DWI sequence as input. A more pronounced enhancement could be observed in the vCE versions including a b1500 input sequence, as compared with the vCE approaches including only b50 and b750. Histopathology confirmed breast carcinoma (NST). **d** Scans show a 60-year-old patient with a non-mass enhancement (NME) in the right breast (21.4 mm area, white arrow) enhancing lightly in the GBCA-enhanced post-contrast subtraction image. The NME does not enhance distinctly in the vCE images that did not include any DWI sequence as input. A more pronounced enhancement could be observed in the vCE versions including a b1500 input sequence, as compared with the vCE training only including b50, b750 DWI sequences. Histopathology confirmed invasive breast cancer. T1w, T1-weighted; T2w, T2-weighted; b50, DWI acquisition with a b-value of 50 s/mm^2^; b750, DWI acquisition with a b-value of 750 s/mm^2^; b1500, DWI acquisition with a b-value of 1500 s/mm^2^; Post-Contrast, subtraction of the second post-contrast phase of a DCE image acquisition; vCE, virtual contrast-enhanced image generated using the respective combination of input sequences

**Fig. 4:**
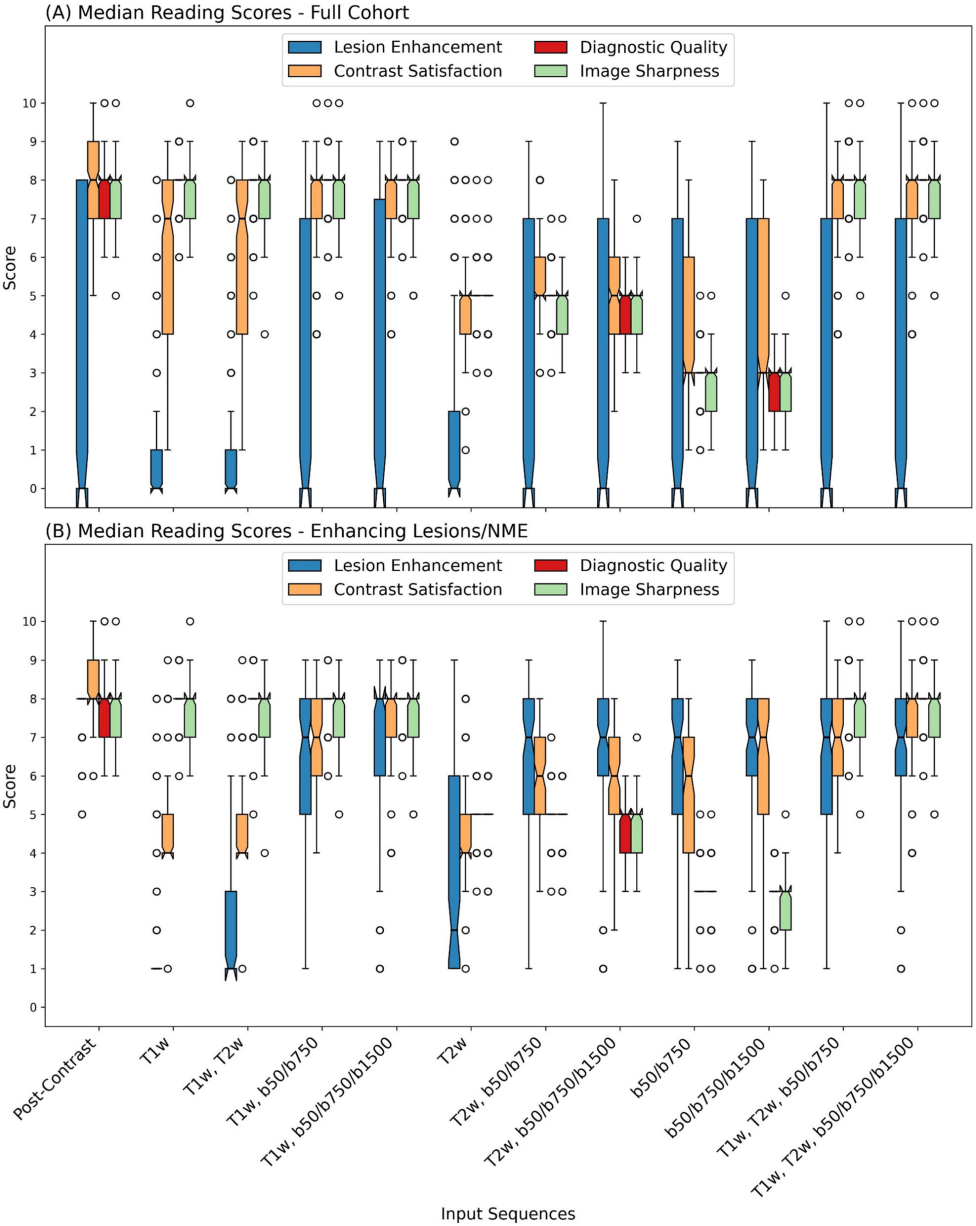
Box plots with notches for the median reading scores among the three readers for the full cohort and the cases with enhancing lesions/NMEs in the GBCA-enhanced post-contrast subtraction image. Significantly lower lesion/NME enhancement could be observed when no DWI acquisitions were included in the input sequence combination. A lower contrast satisfaction score could be noted in the enhancing lesions/NMEs with no b1500 acquisition than with inclusion (e.g., T1w, b50/b750 vs T1w, b50/b705/b1500). All input combinations that did not include a T1w sequence showed a lower score in the diagnostic quality and image sharpness. T1w, T1-weighted; T2w, T2-weighted; b50, DWI acquisition with a b-value of 50 s/mm^2^; b750, DWI acquisition with a b-value of 750 s/mm^2^; b1500, DWI acquisition with a b-value of 1500 s/mm^2^; Post-Contrast, subtraction of the second post-contrast phase of a DCE image acquisition. The notches indicate 95% confidence intervals, and the boxes show the interquartile range

**Fig. 5:**
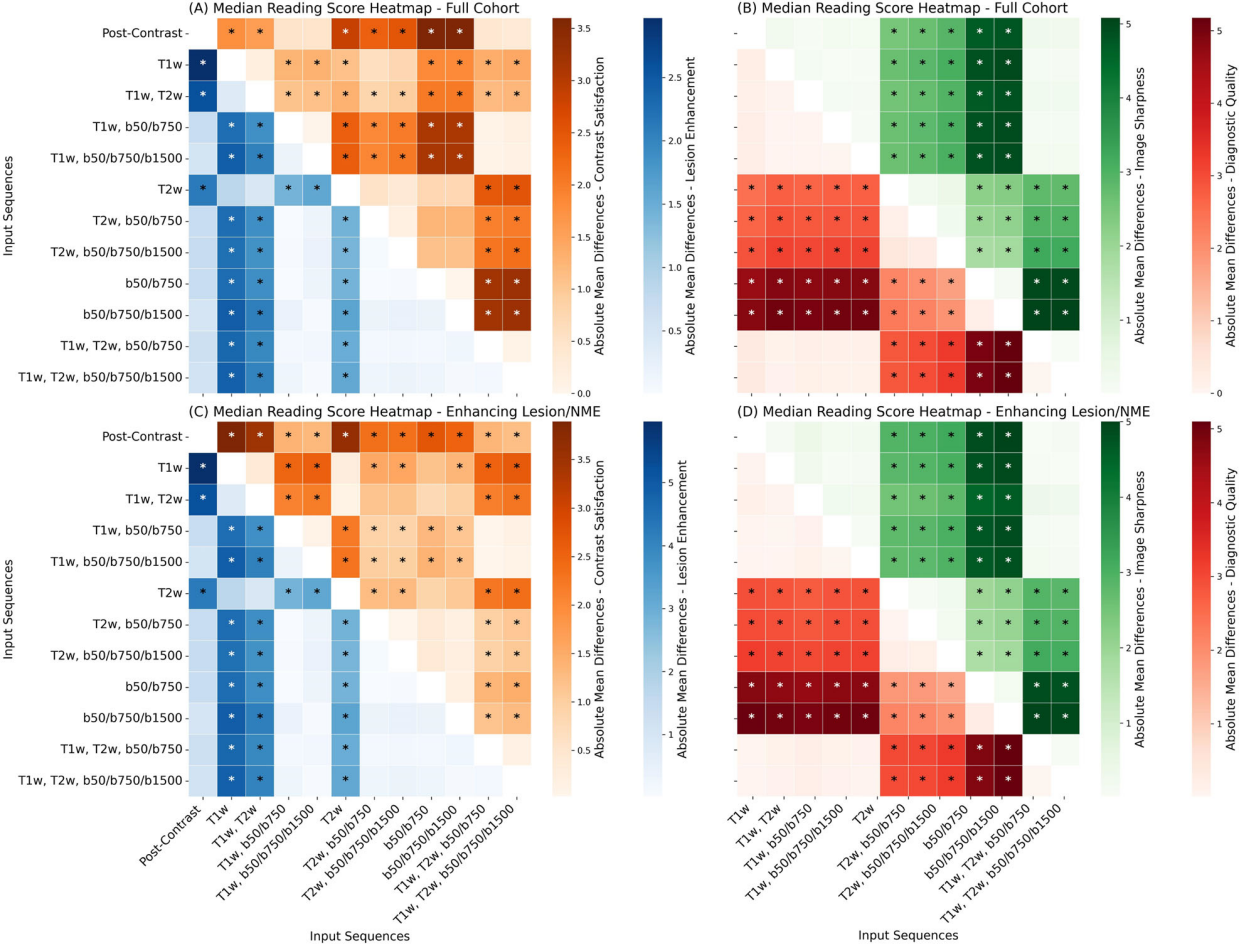
Correlation plot showing the mean absolute differences in the median reading scores for the full cohort (**A**, **B**) and the cases with enhancing lesions/NMEs in the post-contrast subtraction image after GBCA injection (**C**, **D**). Significance of the difference in the median reading score between the respective methods is marked with stars. T1w, T1-weighted; T2w, T2-weighted; b50, DWI acquisition with a b-value of 50 s/mm^2^; b750, DWI acquisition with a b-value of 750 s/mm^2^; b1500, DWI acquisition with a b-value of 1500 s/mm^2^; Post-Contrast, subtraction of the second post-contrast phase of a DCE image acquisition. *p < 0.05, **p < 0.01, ***p < 0.001

Four cases are demonstrated in Fig. 3a–d. Approximately *n* = 93/187 (49.7%) of the independent test cases were identified as showing a mass enhancing lesion (*n* = 61/93, 65.6%) or NME (*n* = 32/93, 34.4%) by at least two readers (further referred to as enhancing lesions/NMEs). The box plots for the qualitative reading are presented in Fig. 4 for the entire patient cohort and the cases with enhancing lesions/NMEs. Significant differences were found in all qualitative scores using the Friedmann test for both the full cohort and cases with enhancing lesions/NMEs (all *p* < 0.001). The post-hoc Nemenyi test results are presented in Fig. 5. The box plots for the qualitative scores among the readers and the reader agreement are presented in Supplementary Figs. 3 and 4.

Table 3 shows the three best-performing input combinations in regard to each of the reading features. Detailed evaluation of each of the reading features is included in the Supplementary Material in the section “Detailed reading results.” Based on this evaluation, it can be noted that the combination of T1w, T2w, b50/b750/1500 was the only combination that appears among the top three combinations for all of the four reading features. At the same time, the combination of T1w, b50/b750/1500 appeared among the top three combinations for three features (Diagnostic Image Quality, Satisfaction with SNR/Contrast and Lesion Enhancement) and provided a higher (not significant; *p* = 1) lesion enhancement score when compared to the T1w, T2w, b50/b750/b1500 combination.

**Table 3:** Top three best-performing combinations in dependency from the reading features

Reading feature	1	2	3
Diagnostic image quality	T1w, T2w, b50/b750	**T1w, T2w, b50/b750/b1500**	T1w, b50/b750/b1500
Image sharpness	T1w, T2w, b50/b750	**T1w, T2w, b50/b750/b1500**	T1w
Satisfaction with SNR/contrast	**T1w, T2w, b50/b750/b1500**	T1w, b50/b750/b1500	T1w, T2w, b50/b750
Lesion enhancement	b50/b750/b1500	T1w, b50/b750/b1500	**T1w, T2w, b50/b750/b1500**

The only input combination appearing for all four reading features is marked with bold

*T1w* T1-weighted, *T2w* T2-weighted, *b50* DWI with a b-value of 50 s/mm^2^, *b750* DWI with a b-value of 750 s/mm^2^, *b1500* DWI with a b-value of 1500 s/mm^2^, *SNR* signal-to-noise ratio

For both input combinations, *n* = 93/93 of the enhancing lesions were identified to provide at least a minimal enhancement score (score 1) by at least two of the three readers on the vCE data. However, for the input combination T1w, b50/b750/1500 *n* = 6/93 (6.5%) lesions were attributed a minimal enhancement score of 1. In contrast, the combination of T1w, T2w, b50/b750/1500, demonstrated *n* = 8/93 lesions (8.6%) with an enhancement score of 1. Detailed characterization of these lesions with minimal enhancement is laid out in the Supplementary Material Section “Evaluation of cases with minimal enhancement.”

## Discussion

This study demonstrated that the performance of vCE breast MRI neural networks significantly depended on the input acquisition sequences used during training. The highest overall performance regarding the conspicuity of lesions/NMEs depicted in GBCA image acquisition both qualitatively and quantitatively was observed when multi-b-value DWI, including an ultra-high b-value, was combined with high-resolution morphologic T1-weighted image acquisition.

Our data further demonstrate the relevance of visual readings and clinical target-focused metrics, since the metrics based on the full image volume were much less influenced by the input variations than the metrics specifically considering the target findings within the breast tissue.

Using non-enhanced MRI acquisitions for deriving vCE images is an emerging field of research in breast MRI [14–18]. Different approaches have been suggested, with many studies focusing on morphologic acquisitions during neural network training. Previous studies have relied on a combination of T1w and T2w image acquisitions to depict anatomical morphology [14–16]. Mueller-Franzes et al [16] conducted the largest study on this vCE approach suggesting a limited capability of vCE imaging using morphologic sequences alone in depicting enhancing lesions, which agrees with our data.

Our result indicates a significant contribution of DWI acquisitions to the ability of vCE imaging to correctly depict enhancing lesions/NMEs. Prior studies supported this finding [17, 18]. Yet, it remained unclear until now whether DWI acquisitions, such as those recommended by EUSOBI [22] or including an ultra-high b-value [21], affect neural networks’ ability to derive vCE images. Our results suggest that adding an ultra-high b-value might enhance lesion/NME conspicuity/enhancement in challenging cases; however, these differences were not statistically significant, given our sample size. Ultra-high b-value DWI scans, when acquired with sufficient image quality, can aid in detecting suspicious breast lesions [21] within healthy fibro-glandular tissue, which typically loses signal beyond a b-value of approximately 1250 s/mm^2^.

The presence of the T2w acquisitions in the input data appeared to affect mostly the image quality and not the ability of vCE to enhance lesions/NMEs. This can be observed in the increase of both qualitative metrics of diagnostic image quality, image sharpness and satisfaction, as well as in the improvement of the quantitative metrics calculated over the whole breast volume (see Fig. 4, Supplementary Table 2 and Supplementary Fig. 2) for data which does include the T2w acquisitions. The lack of impact on the ability to improve the enhancement of lesions/NMEs confirms the observations of Mueller-Franzes et al [16], who indicated that networks which use just T1w and T2w data inputs were not able to provide good lesion enhancement in most cases.

Our study indicates that some lesions show a significantly lower virtual enhancement as compared to GBCA-enhanced subtractions. This was the case even in input combination considered preferable for this task (input sequences: T1w, b50/b750/1500) with about 16.1% of the lesions demonstrating an enhancement score < 50% of the GBCA-enhanced subtractions and *n* = 6/92 lesions (6.5%) demonstrating only a minimal enhancement score of 1—among one malignant lesion. However, analyzing those cases did not reveal a clear association to characteristics of histopathology, enhancement pattern or lesion size—yet a slightly higher scoring variance was observed for smaller lesions. It needs to be considered that our study sample size was not powered to investigate this dependency with sufficient validity. Interestingly, while, as mentioned previously, slightly improving overall image quality, adding the T2w sequence to the input tripled low-enhancing scores in malignant cases potentially suggesting fluid-overweighting in the input to impact the neuronal network, leading to misinterpretation of enhancing lesions towards cystic findings.

The quantitative similarity (SSIM and PSNR) and error metrics (NRMSE and MEDSYMAC) of the full images demonstrated only minor differences compared with the results of the visual readings and focused analyses of the target findings. Notably, even the images trained on T1w images alone provided overall high similarity and low error values in the entire breast volume. However, false negatives regarding lesions/NMEs enhancement were consistently found with this approach by all three readers. Such a situation can be attributed to the fact that suspicious lesions/NMEs constitute a substantially small fraction of the images; hence, during averaging of the similarity and error metrics over the whole breast volume, the missing enhancement does not influence the final quantitative metrics. Our observation that evaluation of synthetic data sets requires the addition of qualitative visual readings due to the limited utility of quantitative metrics only is in agreement with previous studies [28]. Notably, our observation is also in agreement with imaging studies investigating deep learning techniques in other organ regions, where quantitative metrics were shown to not catch all subtle differences between datasets in MRI reconstruction tasks [29], in image quality and artifact evaluations [30], and in image segmentation [31, 32].

Patient characteristics might additively influence the results and limit the generalizability of vCE image generation models. Chung et al [17] exclusively included patients with biopsy-proven invasive breast cancer with a mean lesion size of 24 mm. This cohort matches that in the study by Zhang et al [18], who did not disclose the lesion size in detail. In contrast, Mueller-Franzes et al [16] investigated women undergoing breast MRI for screening with a consecutively smaller median lesion size of about 15 mm. With a median size of all findings of 14.5 mm and a median malignant lesion size of 20.4 mm, our study is in between these two studies. Our study further included a diverse clinical real-life spectrum of women undergoing breast MRI in a tertiary hospital. We did not further select patient subgroups to avoid selection bias, influencing the network and allowing for the inclusion of a natural distribution of lesion sizes, lesion characteristics, and clinical indications for breast MRI.

Given the nascent state of this research field, many questions and challenges must be addressed, including the limitations of our study. While we encompassed two MRI scanner systems and encountered a real-world spectrum of clinical cases in breast MRI across training and test sets, additional research is needed to ascertain the limits of generalizability, robustness, and clinical applicability. This extends to variations in sequence settings, vendors, and field strengths. Moreover, we investigated only a single encoder–decoder neural network architecture, but future studies should evaluate other neural network architecture types, such as other encoder–decoder architectures, GANs, or transformers. Next, diagnostic accuracy was not evaluated; rather, “soft” criteria of lesion/NME conspicuity and image quality were selected, as described in previous studies [16, 17], together with quantitative metrics. Thus, no conclusion could be drawn based on our data about the potential clinical applicability of the approach or about potentially relevant influencing clinical factors, especially with regard to the influence of lesion size or type on lesion visualization.

Larger-scale studies with multicentric designs and blinded readings are therefore necessary to determine the potential clinical application (e.g., in intermediate-risk screening). Further, although a broad spectrum of input sequences including their sub-settings (different b-value combinations) was explored, no conclusion could be drawn about potentially relevant complementary contrasts such as chemical exchange saturation transfer [33–35].

In conclusion, a neural network for generating virtual contrast-enhanced images performed best when fed with a multiparametric unenhanced breast MRI protocol, which included both high-resolution morphologic information and multi-b-value DWI with ultra-high b-value image acquisition. Further research is needed to more comprehensively explore virtual contrast-enhanced approaches before assessing their clinical applicability.

## Supplementary information

ELECTRONIC SUPPLEMENTARY MATERIAL

## Abbreviations

b1500: Diffusion-weighted imaging acquisition with a b-value of 1500 s/mm^2^
b50: Diffusion-weighted imaging acquisition with a b-value of 50 s/mm^2^
b750: Diffusion-weighted imaging acquisition with a b-value of 750 s/mm^2^
CE: Contrast-enhanced
MEDSYMAC: Median symmetrical accuracy
NME: Non-mass-enhancement
NRMSE: Normalized root mean square error
PSNR: Peak signal-to-noise-ratio
SSIM: Structural similarity index
vCE: Virtual contrast-enhanced
