# Supplementary material for: Impact of non-contrast-enhanced imaging input sequences on the generation of virtual contrast-enhanced breast MRI scans using neural network
Source: Eur Radiol. 2024 Oct 25;35(5):2603–16. doi: 10.1007/s00330-024-11142-3 (PMC12021982; doi:10.1007/s00330-024-11142-3)
Supplement: Supplementary file 1 — ELECTRONIC SUPPLEMENTARY MATERIAL [file 330_2024_11142_MOESM1_ESM.docx]

**Impact of Non-Contrast Enhanced Imaging Input Sequences on the Generation of Virtual Contrast-Enhanced Breast MRI Scans** **using Neural Networks**

**ELECTRONIC SUPPLEMENTARY MATERIAL**

**Materials and Methods**

**Literature Review**

A literature review was conducted including peer-reviewed articles/conference abstracts to establish the current knowledge on vCE breast MRI. The primary focus was to identify MRI sequences utilized as input data, specific neural network architectures implemented, and experiments performed for both quantitative and qualitative evaluations of results. The search for relevant articles was performed in major databases, including PubMed, IEEE Xplore, ScienceDirect, and arXiv, as well as for conference abstracts in the International Society for Magnetic Resonance in Medicine annual meetings for the years 2020–2022 and the European Congress of Radiology 2022 using the following targeted search terms: “virtual contrast enhancement,” “synthetic contrast enhancement,” “breast MRI,” “neural networks,” and “generative adversarial network.” The selection criteria were based on the relevance of the studies to our research question, with a focus on original research articles that reported the development and evaluation of neural network-based vCE imaging methods for breast MRI. Based on this investigation, seven different publications and conference abstracts were identified.

Following the identification of the relevant publications, we extracted and compared the details of the employed MRI sequences (e.g., T1-weighted, T2-weighted, and diffusion-weighted imaging), neural network architectures (e.g., encoder–decoder convolutional neural networks or GANs), number of patients (with distributions into training, validation, and test datasets), and quantitative and qualitative evaluation methods.

**Image preprocessing:**

The in-plane resolution and number of slices varied strongly across the image series used in the study. Therefore, all series were resampled to a common in-plane matrix of 448x280. Since the trainings were performed in 2D, the number of slices along the Z-axis was retained as in the original T1w acquisition. The position of the full matrix origin and thus, the field-of-view, was based on the matrix origin of the DWI acquisition. The pixel spacing was based on the T1-weighted acquisition. This preprocessing allows to create a voxel-wise correlation between the different image series. Notably, no motion correction or co-registration of the volumes was performed. The preprocessing was performed using Python (Version 3.8.12) and the SimpleITK framework (Version 2.1.1) using an in-house developed script.
The T1-weighted, T2-weighted and each individual DWI acquisition were z-score normalized, clamped within the range [-1, 15] and scaled to [0, 1]. The GBCA-subtractions of all five time-points were normalized, clamped and scaled to [-1, 1].

**Neural Network Architecture and Training**

A graphical depiction of the network is shown in Supplemental Figure 1. The networks were trained on a dedicated workstation (Linux Ubuntu 20.04, Intel Xeon E5-2698, 2.20 GHz, 48 Cores, 256 GB RAM) using a single Nvidia v100 GPU card with 32 GB RAM. During training, batches of 35 slices were used, and, in each batch, it was ensured that random slices from different examinations were present. The network was trained for 35 epochs without early stopping. The learning rate during the training was set to 10^-3^. The neural network model from the epoch with the lowest value of the loss function over the validation set was used as the final model to generate the image series of the test set.

An adjusted loss function from the work of Chen et al. [1] was used during training. As our data lacked the segmentation of lesions, a combination of the structural similarity index and L1 norm was noted, as presented in the equation below.

$$L\left( y,\hat{y} \right)=\left( 1-\left[ l\left( y,\hat{y} \right) \right]^{\alpha}\cdot\left[ c\left( y,\hat{y} \right) \right]^{\beta}\cdot\left[ s\left( y,\hat{y} \right) \right]^{\gamma} \right)+\sum_{i=0}^{N} |y_{i}-\hat{y}_{i}|$$

The network was implemented using Python (version 3.8.10) via the PyTorch (version 1.9.0) framework.

**Rationale for the Exclusion of data-types**

We did not use ADC maps as input data, as they are prone to errors introduced during ADC calculation. We believe that neural networks themselves can derive the same information from raw DWI data without the need for ADC maps. Additionally, as suggested by Muller-Franzes et al. [2], the simulated low-dose images were not used, as these images are not based solely on native input sequences. The simulated low-dose images require a preprocessing of a contrast-enhanced MRI acquisition, which deviates from our goal of investigating the influence of only native MRI input sequences.

**Segmentation of Target Findings**

The target findings were segmented by a medical student with 2 years of experience in breast MRI research under the supervision of a board-certified radiologist with >10 years of experience using the open-source 3D Slicer Software’s [version 4.11, Fedorov et al. [3]] built-in region draw function. The target findings were selected in consensus based on the clinical routine report and then segmented manually using the original post-contrast images as matrix and the full multiparametric information of the MRI examinations (e.g., in case of non-enhancing lesions such as cysts, in which, however, contours of the cysts can be delineated carefully when shifting co-registered images in between T1-weighted post-contrast subtraction and T2-weighted sequences). The segmentations were performed along the inner border of the target finding, suing the slice depicting the target finding most centrally. In case of multiple target findings, only the largest target finding within the breast volume was segmented. The size of the target finding was measured as the length of the longest axis in the segmented target finding. For the analysis of the target finding regarding the performance metrics, a bounding box around the segmentation was drawn using the skimage Python package. The metrics were then calculated only inside the bounding box.

**Quantitative Metrics Choice**

The literature review (Table 1) showed that the two most commonly used similarity metrics were the SSIM and PSNR. The SSIM was calculated in accordance with the work of Wang et al. [4]. The error metrics selected were the normalized root mean square error (NRMSE) and median symmetrical accuracy (MEDSYMAC). We used the MEDSYMAC instead of the symmetric mean absolute percentage error (SMAPE) or mean absolute percentage error (MAPE), as subtraction images commonly consist of many substantially low values; the SMAPE and MAPE could then potentially overestimate the error, as they are prone to values close to 0 [5].

**Inter-Reader Agreement Evaluation**

Inter-reader agreement between the Likert scale-assessed variables was evaluated using Kendall’s coefficient of concordance.

**Statistical Analysis**

Differences in the ordered variables between the generated vCE images and CE ground truth images were evaluated using the Friedmann’s test, followed by a post-hoc Nemanyi test in significant cases. The resulting p-values were adjusted for multiple comparisons using the Bonferroni method and a p-value of 0.05 was considered as significant. Differences in the quantitative scores were determined both for metrics in the entire breast and segmented findings. Differences in the qualitative scores were evaluated both for all patients and patients among whom an enhancing lesion/NME was identified in the original CE image after a majority voting by all three readers. All statistical analyses were performed using Python (version 3.9.13).

**RESULTS**

**Inter-Reader Agreement Evaluation**

Kendall’s coefficient of concordance showed the highest agreement between the three readers for the lesion enhancement score (mean=0.94±0.01), followed by the satisfaction with image contrast and SNR (mean=0.88±0.04). The image quality and sharpness showed lower but still good agreement between the readers (mean=0.82±0.04 and 0.80±0.04, respectively).

**Detailed Reading Results**

The input sequence combinations not containing a T1w sequence showed significantly lower median values for the overall image quality and image sharpness for both the full cohort and cases with enhancing lesions/NMEs (highest p-vale: 0.012 main manuscript Figure 5A and 5D). The best performing input sequence combination in regards to both image sharpness and the diagnostic image quality was the combination of T1w, T2w, b50/b750.

For the full cohort all combinations which included any DWI acquisition (either b50/b750 or b50/b750/b1500) showed no significant difference (all p-values=1.00) in regards to the lesion/NME conspicuity when compared to original Post-Contrast images (main manuscript Figure 5A). The best performing input combination in this regard was the combination of b50/b750/b1500.

In regards to contrast satisfaction only combination which included both DWI acquisitions and T1w acquisitions showed no significant difference (p=1.0) when compared to the original Post-Contrast images for the full cohort (main manuscript Figure 5A). However, in the subgroup analysis of the enhancing lesions/NME’s it could be noted that a significant difference from the Post-Contrast could be observed for all input combinations (highest p=0.012, main manuscript Figure 5C). The best performing input sequence combination in regards to the contrast satisfaction was the combination of T1w, T2w b50/b750/b1500 for both the full cohort and for the enhancing lesions/NME’s.

The combinations best-performing in regards to quantitative values in the target findings (T1w, b50/b750/b1500) didn’t significantly differ from the original Post-Contrast images in regards to all of the reading features (p=1.0, main manuscript Figure 5).

Both lesion/NME conspicuity and contrast satisfaction improved when T1w image acquisition was used together with the ultra-high b-value (b50/b750/b1500) compared with those when only a low b-value (b50/b750) was utilized, although the difference was not significant (p=1.0 for both with and without inclusion of T2w). Example cases showing such visual improvement of the lesion conspicuity by the inclusion of the b1500 are shown in Figure 3c and d.

**Evaluation of cases with minimal enhancement**

The n=6/93 (6.5%) lesions which were attributed a minimal enhancement score of 1 in the T1w, b50/b750/1500 combination, included n=1 malignant (breast cancer non special type, cT1) and n=5 benign lesions (scar tissue (n=1), mastopathy (n=2), fibroadenoma (n=2). Amongst these n=6 lesions n=5 cases were masses and one case appeared as NME. Lesion size evaluation revealed n=3 of those cases to be under <10 mm of size, n=1 with a size between 10 and 20 mm and n=2 with a size above 20 mm. The n=8/93 lesions (8.6%) with an enhancement score of 1 in the combination of T1w, T2w, b50/b750/1500, included the beforementioned malignant case and two additive malignant cases (both breast cancer cases with an NST) and the beforementioned benign cases. For this input combination among the n=8 lesions showing a minimal enhancement score of 1, n=7 cases were masses and one case appeared as NME. Lesion size evaluation revealed n=3 of those cases to be under <10 mm of size, n=2 with <20mm of size and n=3 with a size above 20 mm.


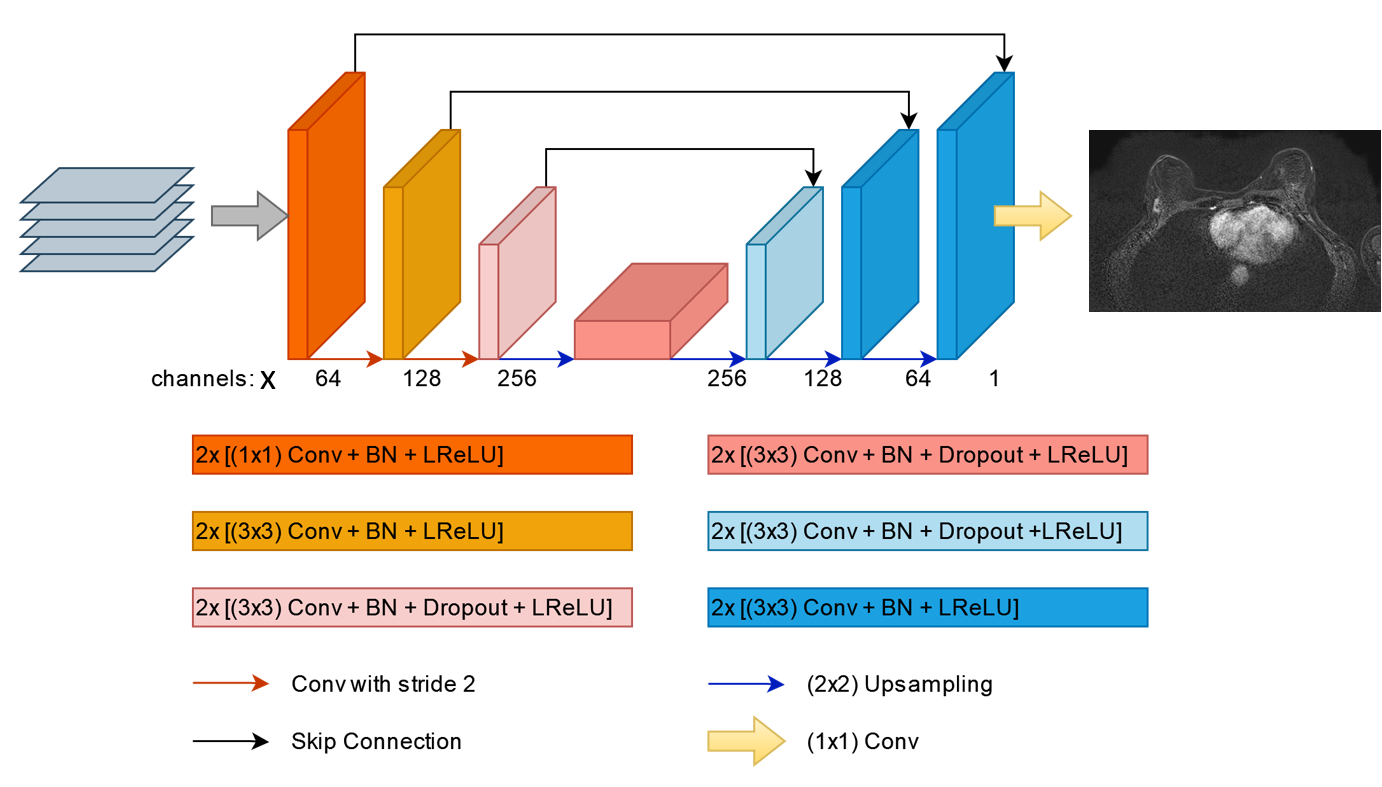
**SUPPLEMENTAL FIGURES**

**Supplemental Figure 1:** A 2D U-net architecture consisted of three encoder and three decoder stages with a bottleneck layer between the deepest encoder and decoder stages followed by an output stage. The number of the networks input channels (x) depends on the specific input sequence combination.

Each encoder and decoder stage consisted of two convolutional layers with a convolutional kernel size of 3, followed by batch normalization (BN) and leaky rectified linear unit (LReLU) activation function. The encoder and decoder stages connected to the bottleneck layer had additional dropout layers between the batch normalization layer and the activation function layer. The down- and up-sampling of the spatial size and feature maps was performed via a 2×2 convolution and a transposed 2×2 convolution with a stride of 2, respectively. The initial encoder stage, was set to generate 64 features, resulting in a maximal feature size of 512. The output layer consisted of 1×1 convolution layer, reducing the number of output channels to 1, and was followed by a tanh activation function to map the output results to [−1,1].

**
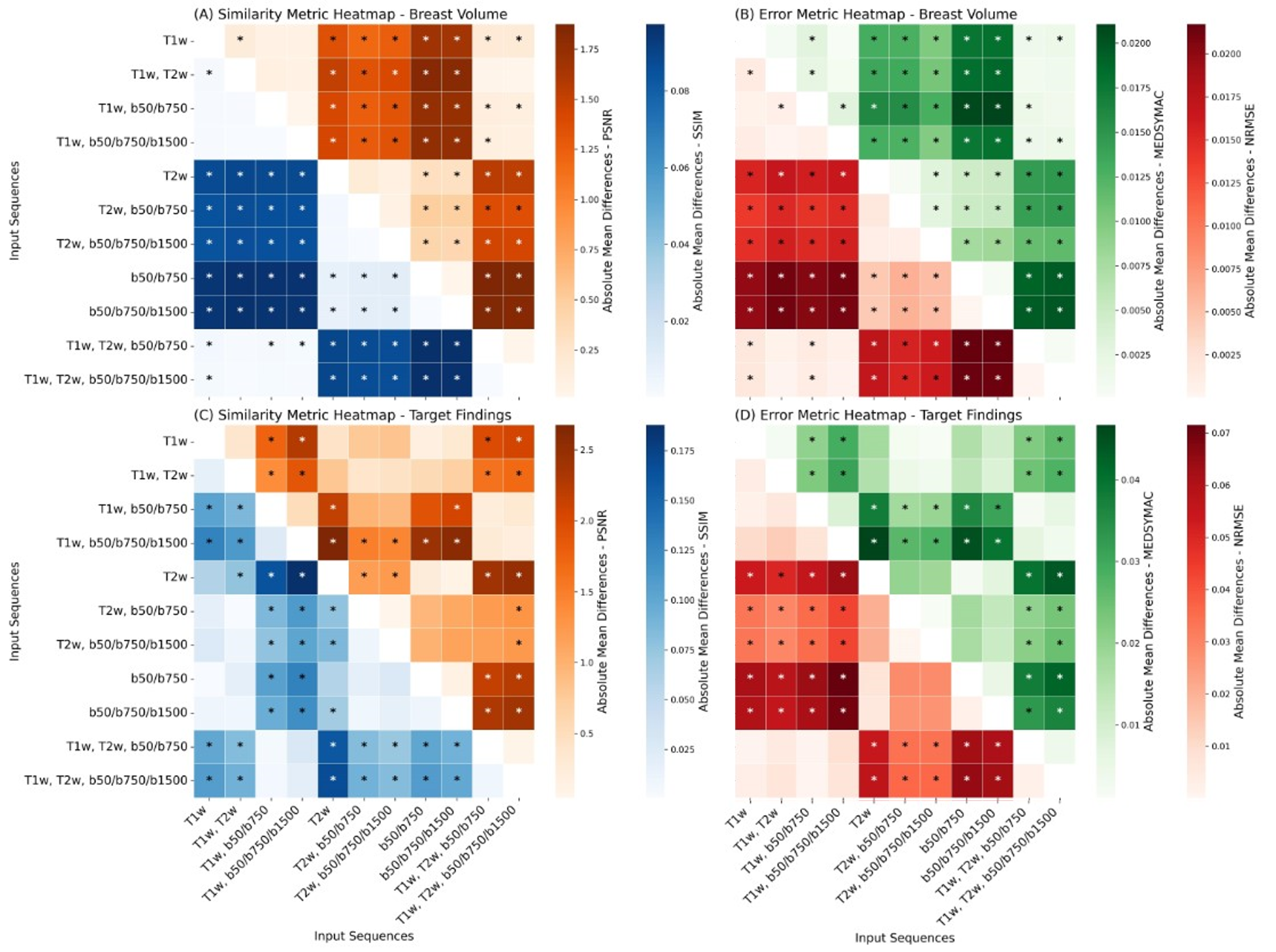
**

**Supplement Figure 2:** Correlation plot showing the mean absolute differences in the median reading scores for the quantitative similarity (a and c) and error (b and d) metrics in the entire breast volume (a and b) and target findings (c and d). Significant differences in the mean of the respective metrics between the different input combination are marked with * (^*^p<.05, ^**^p<.01, ^***^p<.001). T1w=T1-weighted, T2w=T2-weighted, b50=DWI acquisition with a b-value of 50 s/mm^2^, b750=DWI acquisition with a b-value of 750 s/mm^2^, b1500=DWI acquisition with a b-value of 1500 s/mm^2^, Post-Contrast=subtraction of the second post-contrast phase of a DCE image acquisition. ^*^p<.05, ^**^p<.01, ^***^p<.001 Target findings refer to the findings within the examination that could be both benign or malignant and non-mass or mass enhancement as well as findings not enhancing (e.g., cysts) but morphologically delineated from healthy fibro-glandular tissue.

**
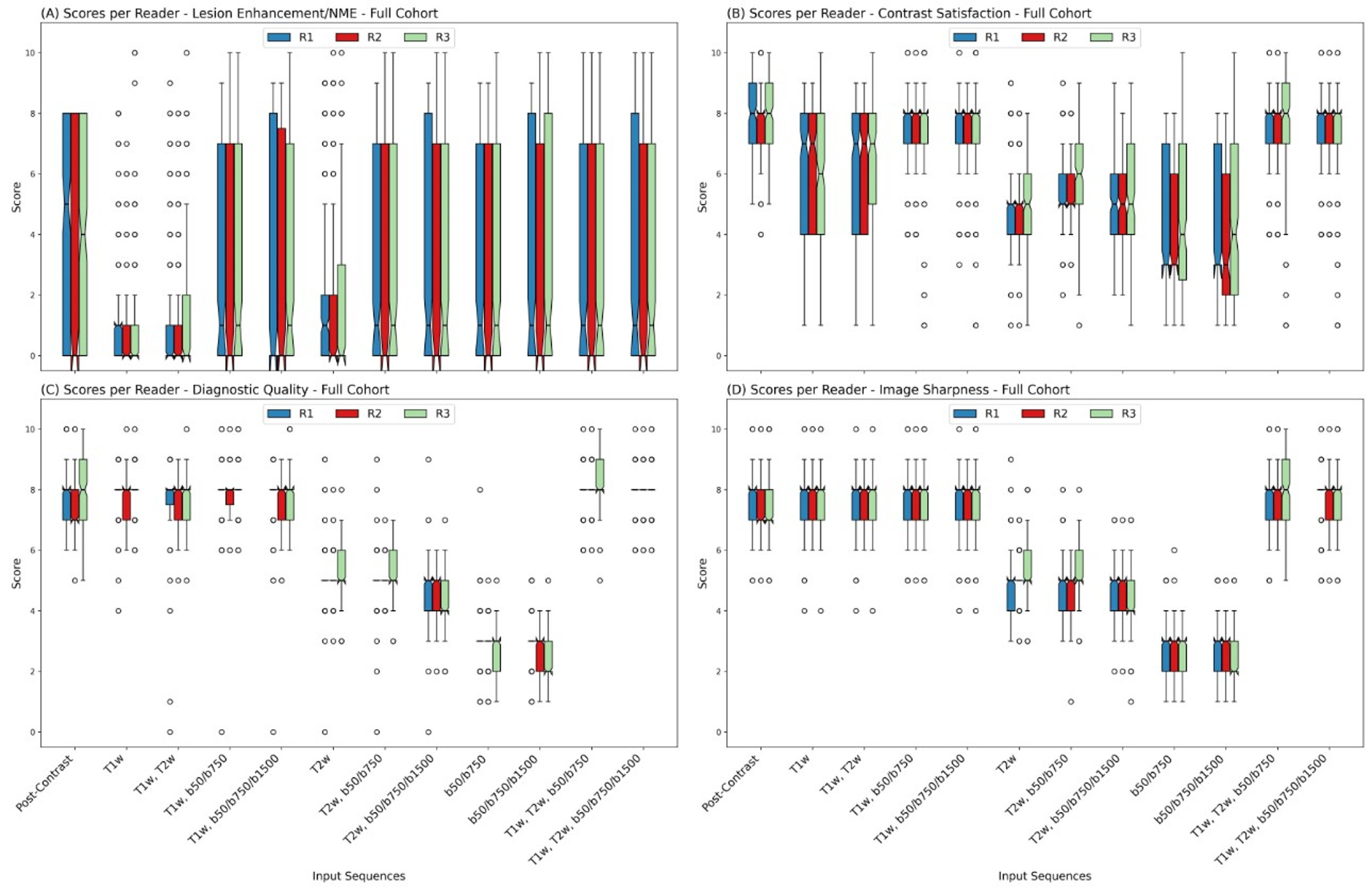
**

**Supplement Figure 3:** Boxplots with notches showing individual qualitative evaluation scores among the three readers (R1, R2, and R3) for the full patient cohort. Notches indicate the 95% confidence intervals, boxes indicate the interquartile range and circles show the outlier scores. A significant drop in the ability to enhance lesions/NMEs could be observed for all three readers when no DWI acquisition was present in the input sequence combination (A). A significant drop in the diagnostic quality and image sharpness score could be noted (C and D) when no T1w acquisition was present in the input sequence combination for all three readers. A significant drop in the contrast satisfaction could be observed when either the T1w image acquisition or DWI acquisition was missing in the input sequence combination (B) for all three readers. T1w=T1-weighted, b50=DWI acquisition with a b-value of 50 s/mm^2^, b750=DWI acquisition with a b-value of 750 s/mm^2^, b1500=DWI acquisition with a b-value of 1500 s/mm^2^, NME=non-mass-enhancement


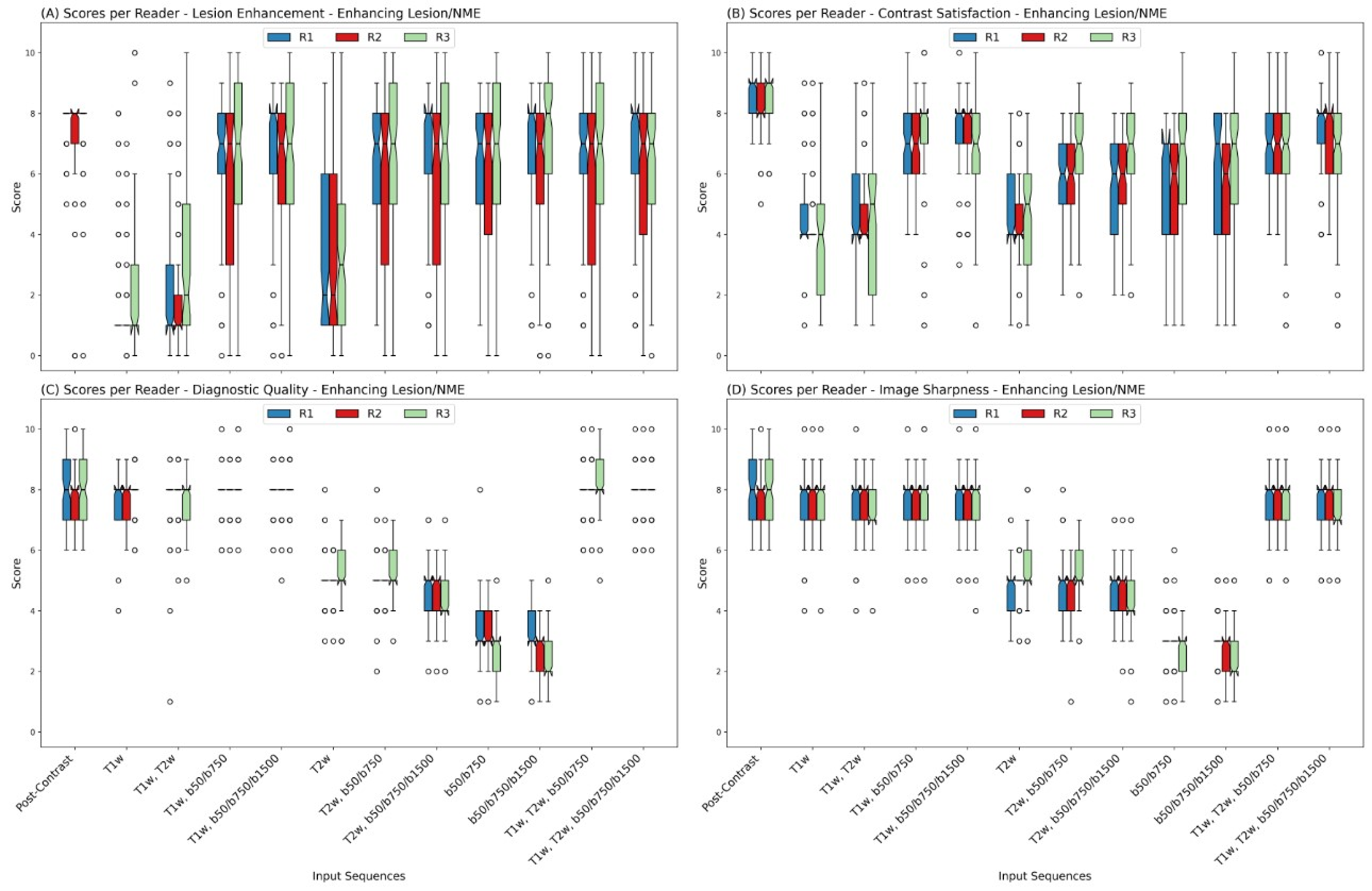


**Supplement Figure 4:** Boxplots with notches showing individual qualitative evaluation scores among the three readers (R1, R2, and R3) for only the cases with enhancing lesions/NMEs in the original post-contrast subtraction image after GBCA injection. Notches indicate 95%-confidence intervals, and boxes indicate the interquartile range, circles indicate outliers. A significant drop in the ability to enhance lesions/NMEs could be observed for all three readers when no DWI acquisition was present in the input sequence combination when compared with the original post-contrast image (A). A significant drop in the diagnostic quality and image sharpness score could be noted when no T1w image acquisition was present in the input sequence combination for all three readers (C and D). For two of the three readers, a significant drop in the contrast satisfaction could be observed when either the T1w image acquisition or DWI acquisition was missing in the input sequence combination (B) when compared to the original Post-Contrast image. Further, in comparison to the evaluation of original Post-Contrast images, for all three readers, an overall lower score of contrast satisfaction could be noted even for input combinations that included both T1w image acquisition and all three DWI acquisitions (B). T1w=T1-weighted, b50=DWI acquisition with a b-value of 50 s/mm^2^, b750=DWI acquisition with a b-value of 750 s/mm^2^, b1500=DWI acquisition with a b-value of 1500 s/mm^2^, NME=non-mass-enhancement

| **Supplement Table 1: Literature Review** | | | | | | | |
| --- | --- | --- | --- | --- | --- | --- | --- |
| Study | No. of Examinations^*^ | Neural Network Architecture | Input Sequences | Field Strength | Quantitative Evaluation | Qualitative Evaluation | Readers |
| Wang et al. [6] | 45/31/21 | GAN–enhance border lifelike synthesize model | T1w | 3.0 | SSIM, PSNR, MSE, and MAE | Satisfaction with the synthesized vCE images in side-by-side comparison with CE images  Assessment of the diagnostic value with three reading protocols including vCE, CE, or DCE series in addition to T1w, T2w, and DWI sequences | 3 |
| Kim et al. [7] | 230/21/55 + 140 external test subjects | GAN–tumor-attentive segmentation-guided GAN | T1w | 1.5, 3.0 (Dataset 1), and 3.0 (Dataset 2) | SSIM, PSNR, NRMSE, and  Pearson cross-correlation | Intensity profiles along the vertical direction of the tumor center  Error map generation  No image reading performed | - |
| Muller-Franzes et al. [2] | 9551/0/200 | GAN – Pix2PixHD | T1w, T2w, simulated low dose | 1.5 | SSIM, PSNR, MSE, and MAE | Determination if readers are able to identify a synthetic image  Satisfaction with the synthesized vCE images in side-by-side comparison with CE images  Comparison of conspicuity of enhancing lesions in side-by-side comparison between vCE and CE images | 2 |
| Sikka et al. [8] | 129/16/16 | Encoder–decoder architecture–2D residual attention U-net | T1w | 1.5 | SSIM, PSNR, Pearson r correlation, and Spearman r correlation | No reading performed | - |
| Liebert et al. [9] | 377/81/76 | Encoder–decoder architecture–2D U-net | T1w, T2w, DWI (b-values: 50, 750, and 1500 s/mm^2^) | 3.0 | No quantitative evaluation performed | Diagnostic performance: sensitivity, specificity, and accuracy | 1 |
| Chung et al. [10] | 86/15 with 7-fold cross validation | Encoder–decoder architecture–GadNet | T1w, T1w Fat-Saturated, T2w, DWI (b-values: 0 and 600 s/mm^2^), ADC | 1.5 and 3.0 | SSIM, neighborhood cross-correlation, HMI, NRMSE, SMAPE, MEDSYMAC, and log accuracy ratio,  DICE coefficient comparing segmentations  Lesion sizes | Comparison with the synthesized vCE images in side-by-side comparison with CE images  Comparison whether the lesion enhanced correctly in side-by-side comparison between vCE and CE images  Evaluation of the quality of both vCE and CE images | 4 |
| Zhang et al. [11] | 612/153 | Encoder–decoder architecture | T1w+DWI (b-values: 0, 150, 800, and 1500 s/mm^2^) | 3.0 | SSIM, PSNR, and NMSE | No reading performed | - |
| ^*^No. refers to the number of examinations in the training/validation/test sets.  GAN=generative-adversarial-network, Pix2PixHD=conditional GAN type, CE= contrast enhanced, T1w=T1-weighted, T2w=T2-weighted, DWI= diffusion weighted, ADC=apparent diffusion coefficient, vCE= virtual contrast-enhanced, SSIM= structural similarity index, PSNR= peak signal-to-noise-ratio, NRMSE=normalized root mean square error, MEDSYMAC=median symmetrical accuracy, MSE=mean square error, MAE=mean absolute error, HMI=histogram mutual information, NMSE= normalized mean square error, SMAPE=Symmetric mean absolute percentage error, DICE - Dice-Sørensen coefficient, | | | | | | | |

| **Supplement Table 2: Quantitative Performance of the Different Network Input Combinations** | | | | | |
| --- | --- | --- | --- | --- | --- |
| Input Combination | Tissue | SSIM (↑) | PSNR [dB] (↑) | NRMSE [%] (↓) | MEDSYMAC [%] (↓) |
| T1w | BV | 86.91±2.58 | 24.18±1.82 | 8.91±1.20 | 2.08±0.95 |
|  | TF^*^ | 50.25±22.84 | 16.77±6.10 | 20.05±6.63 | 13.92±5.19 |
| T1w+T2w | BV | 87.06±2.57 | 24.33±1.79 | 8.77±1.17 | 2.02±0.92 |
|  | TF^*^ | 51.94±22.80 | 17.15±6.17 | 20.41±7.49 | 14.12±6.28 |
| T1w, b50/b750 | BV | 86.98±2.60 | 24.25±1.75 | 8.86±1.18 | **1.78±0.85** |
|  | TF^*^ | 60.63±20.71 | 18.51±5.25 | 19.82±7.46 | 11.88±5.58 |
| T1w, b50/b750/b1500 | BV | 87.00±2.55 | 24.26±1.83 | 8.79±1.20 | 2.09±0.92 |
|  | TF^*^ | **63.08±20.16** | **19.02±5.24** | **19.06±7.83** | **11.01±5.07** |
| T2w | BV | 78.18±5.13 | 22.82±1.70 | 10.44±1.32 | 3.38±1.26 |
|  | TF^*^ | 44.29±21.58 | 16.34±5.28 | 25.43±7.89 | 15.69±5.72 |
| T2w, b50/b750 | BV | 78.41±5.07 | 23.00±1.75 | 10.28±1.37 | 3.36±1.28 |
|  | TF^*^ | 52.02±21.43 | 17.54±4.85 | 23.37±8.06 | 13.69±5.63 |
| T2w, b50/b750/b1500 | BV | 78.44±5.09 | 22.92±1.75 | 10.36±1.39 | 3.07±1.23 |
|  | TF^*^ | 52.90±21.21 | 17.58±4.97 | 23.36±8.10 | 13.82±5.62 |
| b50/b750 | BV | 77.38±4.92 | 22.50±1.68 | 10.90±1.32 | 3.85±1.35 |
|  | TF^*^ | 50.16±22.74 | 16.59±4.81 | 26.21±8.32 | 15.51±5.59 |
| b50/b750/b1500 | BV | 77.41±4.93 | 22.51±1.73 | 10.89±1.36 | 3.89±1.33 |
|  | TF^*^ | 51.17±22.79 | 16.45±4.90 | 26.01±9.84 | 14.98±5.67 |
| T1w, T2w, b50/b750 | BV | **87.12±2.58** | **24.38±1.80** | **8.73±1.18** | 1.94±0.87 |
|  | TF^*^ | 60.24±20.46 | 18.75±5.55 | 19.94±8.39 | 11.70±5.60 |
| T1w, T2w, b50/b750/b1500 | BV | 87.10±2.55 | 24.36±1.78 | 8.74±1.17 | 1.91±0.87 |
|  | TF^*^ | 61.11±20.24 | 18.16±5.57 | 19.67±8.21 | 11.46±5.17 |
| Arrows indicate whether higher (↑) or lower (↓) values of the respective metric indicate better performance. Bold marked values indicate the best performance for the respective tissue. Underscored values indicate worst performance for respective tissue type and metric across all input combinations.  T1w=T1-weighted, T2w=T2-weighted, b50=diffusion-weighted imaging with a b-value of 50 s/mm^2^, b750=diffusion-weighted imaging with a b-value of 750 s/mm^2^, b1500=diffusion-weighted imaging with a b-value of 1500 s/mm^2^, BV=breast volume, TF – Target findings, SSIM=structural similarity index, PSNR=peak signal-to-noise ratio, dB=decibel, NRMSE=normalized root mean square error, MEDSYMAC=median symmetrical accuracy  ^*^Target findings refer to the findings within the examination that could be both benign or malignant and non-mass or mass enhancement as well as findings not enhancing (e.g., cysts) but morphologically delineated from healthy fibro-glandular tissue. | | | | | |

**REFERENCES**

1 Chen C, Raymond C, Speier B et al (2021) Synthesizing MR Image Contrast Enhancement Using 3D High-resolution ConvNets. arXiv preprint arXiv:210401592

2 Muller-Franzes G, Huck L, Tayebi Arasteh S et al (2023) Using Machine Learning to Reduce the Need for Contrast Agents in Breast MRI through Synthetic Images. Radiology. 10.1148/radiol.222211:222211

3 Fedorov A, Beichel R, Kalpathy-Cramer J et al (2012) 3D Slicer as an image computing platform for the Quantitative Imaging Network. Magn Reson Imaging 30:1323-1341

4 Wang Z, Bovik AC, Sheikh HR, Simoncelli EP (2004) Image quality assessment: from error visibility to structural similarity. IEEE transactions on image processing 13:600-612

5 Morley SK, Brito TV, Welling DT (2018) Measures of model performance based on the log accuracy ratio. Space Weather 16:69-88

6 Wang P, Nie P, Dang Y et al (2021) Synthesizing the First Phase of Dynamic Sequences of Breast MRI for Enhanced Lesion Identification. Front Oncol 11:792516

7 Kim E, Cho H-H, Kwon J, Oh Y-T, Ko ES, Park H (2022) Tumor-Attentive Segmentation-Guided GAN for Synthesizing Breast Contrast-Enhanced MRI Without Contrast Agents. IEEE Journal of Translational Engineering in Health and Medicine 11:32-43

8 Sikka D, Zhu N, Liu C, Small S, Guo J Predicting Gadolinium Contrast Enhancement for Structural Lesion Analysis using DeepContrast.

9 Liebert A, Schreiter H, Kapsner LA et al (2022) Virtual abbreviated contrast enhanced MRI for breast cancer diagnostics – initial experienceEuropean Congress of Radiology, Vienna

10 Chung M, Calabrese E, Mongan J et al (2022) Deep Learning to Simulate Contrast-enhanced Breast MRI of Invasive Breast Cancer. Radiology. 10.1148/radiol.213199:213199

11 Zhang T, Han L, D’Angelo A et al (2023) Synthesis of Contrast-Enhanced Breast MRI Using T1- and Multi-b-Value DWI-Based Hierarchical Fusion Network with Attention Mechanism. Springer Nature Switzerland, Cham, pp 79-88
